# Serum, but Not Saliva, CXCL13 Levels Associate With Infiltrating CXCL13+ Cells in the Minor Salivary Gland Lesions and Other Histologic Parameters in Patients With Sjögren’s Syndrome

**DOI:** 10.3389/fimmu.2021.705079

**Published:** 2021-08-17

**Authors:** Loukas Chatzis, Andreas V. Goules, Ioanna E. Stergiou, Michael Voulgarelis, Athanasios G. Tzioufas, Efstathia K. Kapsogeorgou

**Affiliations:** ^1^Department of Pathophysiology, School of Medicine, National and Kapodistrian University of Athens, Athens, Greece; ^2^Institute for Autoimmune Systemic and Neurological Diseases, Athens, Greece

**Keywords:** Sjögren’s syndrome, non-Hodgkin’s lymphoma, CXCL13 chemokine, serum, saliva, minor salivary gland

## Abstract

Recent studies suggest that elevated CXCL13 serum levels in patients with primary Sjögren’s syndrome (pSS) associate with minor salivary gland (MSG) histologic features, disease severity, as well as high-risk status for non-Hodgkin lymphoma (NHL) development and NHL itself. In contrast, limited discriminative value of CXCL13 saliva levels has been reported. Prompt by these reports, we sought to validate the clinical utility of CXCL13 by investigating potential correlations of serum and saliva levels with MSG histopathologic [including CXCL13+-cell number, severity of infiltrates and germinal center (GC) formation], serologic and clinical parameters, as well as NHL. CXCL13 levels were evaluated in paired serum and saliva specimens of 45 pSS patients (15 with NHL; pSS-associated NHL: SSL), 11 sicca-controls (sicca-complaining individuals with negative MSG biopsy and negative autoantibody profile), 10 healthy individuals (healthy-controls) and 6 non-SS-NHLs. CXCL13+-cells were measured in paired MSG-tissues of 22 of pSS patients studied (including 7 SSLs) and all sicca-controls. CXCL13 serum levels were significantly increased in pSS and SSL patients compared to sicca- and healthy-controls and were positively correlated with the CXCL13+-cell number and biopsy focus-score. Serum CXCL13 was significantly higher in pSS patients with GCs, rheumatoid factor, hypocomplementemia, high disease activity, NHL and in high-risk patients for NHL development. CXCL13 saliva levels were significantly increased in SSL patients (compared to non-SS-NHLs), patients with GCs and in high-risk for NHL patients. Univariate analysis revealed that CXCL13 serum, but not saliva, levels were associated with lymphoma, an association that did not survive multivariate analysis. Conclusively, our findings confirm that serum, but not saliva, levels of CXCL13 are associated with histologic, serologic and clinical features indicative of more severe pSS.

## Introduction

Non-Hodgkin’s lymphomas (NHL) of B cell origin are often developed in the setting of autoimmune diseases (1). Primary Sjögren’s syndrome (pSS) is the disorder with the highest prevalence of NHL (5-10%) among autoimmune diseases (2). In fact, NHL represents the major adverse outcome of the disease, affecting both morbidity and mortality (3–5). Although the underlying pathogenetic mechanisms of SS-related lymphomagenesis are not defined, it is considered a multistep, antigen-driven process arising from incessant chronic B cell activation in the inflammatory lesions (6, 7). Indeed, the vast majority of SS- related NHLs are mucosa-associated lymphoid tissue (MALT) lymphomas, located in the salivary glands and their presence is heralded by intense inflammatory responses, as attested by the degree of infiltration, certain inflammatory cells, such as macrophages, and organization of the lymphocytic infiltrates to ectopic germinal centers (eGC) within the affected minor salivary glands (MSG) (8).

CXCL13 (C-X-C motif chemokine ligand 13) is a chemokine expressed by follicular dendritic cells (FDC), stromal cells, monocytes and macrophages, primarily in secondary lymphoid organs, such as the liver, spleen and lymph nodes (9, 10). Through interaction with the G protein-coupled chemokine receptor CXCR5, which is expressed on B and T follicular helper cells, CXCL13 holds a vital role for the development and organization of lymphoid tissues (9–13). CXCL13 has been mechanistically linked to several disorders, including autoimmune diseases and hematologic malignancies (14–23). In pSS, CXCL13 expression has been implicated in SS pathogenesis, including the formation of eGCs in the MSG lesions and the related process leading to lymphomagenesis (7, 24, 25). Increased CXCL13 serum levels have been associated with NHL, pSS disease activity and MSG histologic features, whilst in a recent study they were linked to increased lymphoma risk (26–30). Interestingly, serum, but not saliva, levels of CXCL13 were found elevated in Asian-Indian pSS patients (31), questioning whether elevated CXCL13 serum levels in patients with extended and/or organized to eGCs inflammatory MSG lesions originate from the affected glands.

Prompted by these findings, we sought to validate the clinical utility of CXCL13 in pSS by examining its expression in paired samples of serum, saliva and MSG biopsies from patients with pSS (with or without NHL), sicca-controls, and healthy individuals. In addition, we investigated possible associations with various histologic, serologic and clinical disease parameters, which have been previously identified as adverse prognostic factors for NHL development, including severity of MSG autoimmune infiltrates and eGC formation, high EULAR SS disease activity index (ESSDAI) score, salivary gland enlargement (SGE), purpura, vasculitis, leukopenia, cryoglobulinemia, hypocomplementemia, autoantibodies against Ro/La, and rheumatoid factor (3, 4, 8, 32–37).

## Materials and Methods

### Patients

Paired samples of serum and unstimulated saliva were obtained at the same time from forty-five pSS patients (38, 39), of whom fifteen had NHL (SSL subgroup), eleven sicca-complaining individuals with no infiltrates in diagnostic MSG biopsy and negative autoantibody profile (sicca-controls; SC subgroup), ten healthy controls (HC subgroup) and six patients with non-SS associated NHL [NHL subgroup; two with diffuse large B-cell lymphoma (DLCBL) and one each with primary mediastinal large B-cell lymphoma, follicular lymphoma, Hodgkin lymphoma and Richter’s transformation in chronic lymphocytic leukemia (CLL)]. The characteristics and treatment of the pSS patients and controls included in the study are summarized in **Table 1**.

**Table 1:** Characteristics of the patients studied.

Features		Controls			SS patients	
		Healthy *(n=10)*	Sicca *(n=11)*	NHL *(n=6)*	SS *(n=30)*	SSL *(n=15)*
**General**	Age (years), median (range)	48 (38-72)	50 (43-78)	59.5 (34-84)	60 (27-79)	70 (53-78)
	Men/women	0/10	2/9	4/2	3/27	1/14
	Disease Duration (years), median (range)	NA	NA	0.5 (0.5-8.0)	10 (0.25-34.0)	17.0 (4-37.0)
**Histological** *(MSG biopsy)*	Biopsy focus score *(number of lymphocytic foci/4mm^2^)*, median (range)	NA	0 (0.0-0.5)	NA	2.40 (1.0-10.44)	3.33 (1.0-10.0)
	Tarpley biopsy score, median (range)	NA	0	NA	2 (1-3)	3 (1-3)
	Germinal center formation “No,(%)”	NA	0 (0)	NA	8 (26.7)	4 (26.7)
**Clinical**	Arthralgias “No,(%)”	NA	1 (9)	NR	21 (70.0)	13 (86.7)
	Arthritis “No,(%)”	NA	0 (0)	NR	4 (13.3)	3 (20.0)
	SG enlargement (SGE) “No,(%)”	NA	0 (0)	NA	11 (36.7)	9 (60.0)
	Raynaud’s phenomenon “No,(%)”	NA	0 (0)	NR	7 (23.3)	7 (46.7)
	Parenchymal organ involvement “No,(%)”	NA	NA	NA	5 (16.7)	4 (26.7)
	*Lung involvement “No,(%)”*	NA	NA	NA	*2 (6.7)*	*4 (26.7)*
	*Renal involvement “No,(%)”*	NA	NA	NA	*0 (0)*	*0 (0)*
	*Liver involvement “No,(%)”*	NA	NA	NA	*3 (10.0)*	*0 (0)*
	Indicative of vasculitic involvement “No,(%)”	NA	NA	NA	1 (3.3)	6 (40.0)
	*Palpable purpura “No,(%)”*	NA	NA	NR	*1 (3.3)*	*4 (26.7)*
	*Vasculitis (%)”No,(%)”*	NA	NA	NR	*0 (0.0)*	*0 (0.0)*
	*Glomerulonephritis “No(%)*	NA	NA	NR	*0 (0)*	*0 (0)*
	*Peripheral neuropathy “No,(%)”*	NA	NA	NR	*1 (3.3)*	*2 (13.3)*
	ESSDAI score, median (range)	NA	NA	NA	3.5 (0-15)	19 (12-25)
**Laboratory**	Anti-Ro/SSA and/or La/SSB positive “No,(%)”	0 (0)	0 (0)	NA	25 (83.3)	13 (86.7)
	*Anti-Ro/SSA positive “No,(%)”*	*0 (0)*	0 (0)	NA	*25 (83.3)*	*13 (86.7)*
	*Anti-La(SSB) positive “No,(%)”*	*0 (0)*	0 (0)	NA	*13 (43.3)*	*8 (53.3)*
	Rheumatoid Factor positive “No,(%)”	0*(0)*	0 (0)	NA	15 (50.0)	13 (86.7)
	C3-levels, median (range)	NR	NR	NR	111.5 (53-160)	102.0 (86-123)
	C4-levels, median (range)	NR	NR	NR	20.5 (7.0-45.6)	14 (1.0-22.4)
	*C4- hypocomplementemia “No,(%)”*	NR	NR	NR	*7 (23.3)*	*10 (66.7)*
	Cryoglobulinemia “No,(%)”	NA	NA	NR	0 (0.0)	5 (33.3)
	Hypergammaglobulinemia “No(%)”	NA	NA	NR	12 (40.0)	5 (33.3)
	Leukopenia “No,(%)”	NA	NA	NR	1 (3.3)	1 (6.7)
**Treatment**	Steroids, “No(%)”	NA	NA	0	2 (6.7)	0
	Hydroxychloroquine, “No(%)”	NA	NA	0	1 (3.3)	0
	Pilocarpine, “No(%)”	NA	NA	0	2 (6.7)	0
	Hydroxychloroquine & pilocarpine, “No(%)”	NA	NA	0	2 (6.7)	0
	Steroids, pilocarpine & hydroxychloroquine, “No(%)”	NA	NA	0	2 (6.7)	0
	Azathioprine, “No(%)”	NA	NA	0	1 (3.3)	0
	Hydroxychloroquine & methotrexate, “No(%)”	NA	NA	0	2 (6.7)	0
*NHL-related treatment administrated prior to sampling*						
	Anti-CD20 *(median 4years before sampling; range 2-8)*, No(%)	NA	NA	0	0	7 (46.6)
	R-CHOP *(DLBCL-pSS patient 7years before sampling)*, No(%)	NA	NA	0	0	1 (6.7)

NA, not applicable.

NR, not recorded.

R-CHOP, Rituximab, Cyclophosphamide, Doxorubicin hydrochloride, Vincristine, Prednisolone.

In 22 of 45 pSS patients (seven with NHL) and all sicca-controls, paired MSG biopsy specimens were available, and the expression of CXCL13 was examined immunohistochemically. The pSS patients without evidence of NHL at the time of serum, saliva and MSG sampling (SS subgroup, n=30) included twenty-three low-risk (median follow-up time 3.7 years, range: 0.0-23.3 years) and seven high-risk (median follow-up time 1.7 years, range: 0.0-3.0 years) for future lymphoma development as defined previously (40). Briefly, patients expressing two or less of the following risk factors, including salivary gland enlargement, lymphadenopathy, Raynaud phenomenon, anti-Ro/SSA or/and anti-La/SSB autoantibodies, rheumatoid factor positivity, monoclonal gammopathy, and C4 hypocomplementemia, were characterized as low-risk, whereas those with three or more as high-risk to develop lymphoma. The pSS patients without evidence of NHL at the time of serum, saliva and MSG sampling (SSwo subgroup) were further classified according to lesion severity as arbitrarily defined (33) by focus (FS) and Tarpley (TS) biopsy scores (mild: FS:1-1.79, TS:1, intermediate: FS:1.8-3.5, TS:2 and severe: FS: 3.6-10.44, TS: 3-4). This group included ten patients with mild (median FS: 1), twelve with intermediate (median FS: 2.4) and eight with severe (median FS: 4.5) lesions at MSGs. The SSL subgroup consisted of twelve MALT lymphomas (two located at parotid glands and the rest at MSGs) and one each with DLCBL, follicular lymphoma and CLL. Sampling was performed at SSL diagnosis in one patient, and on 4.5 years (median; range: 0.5-18 years) after SSL diagnosis in the rest. CXCL13 levels were also evaluated in sequential sera of six additional pSS patients obtained before NHL onset (pre-lymphoma; median time to lymphoma diagnosis 3.63 years, range 3.0-9.33 years) and on lymphoma onset (median age on pre-lymphoma serum sampling 56 years, range: 29-67) to study the CXCL13 kinetics towards lymphomagenesis.

Medical records were retrospectively evaluated for various clinical, laboratory and histological parameters of SS and lymphoma, including MSG biopsy scoring, ESSDAI, arthralgias, arthritis, Raynaud’s phenomenon, SGE, palpable purpura, vasculitis, lung involvement, as attested by pulmonary-function tests and X-ray and/or computed-tomography scans, renal involvement (persistent proteinuria/glomerular hematuria and verification by renal biopsy), liver involvement (liver-biopsy indicative of primary biliary cirrhosis), peripheral neuropathy as attested by nerve-conduction studies, anti-Ro/SSA and/or anti-La/SSB autoantibodies, rheumatoid factor, hypocomplementemia (C4<16mg/dL and C3<75mg/dL), hypergammaglobulinemia (IgG gammaglobulins>2g/L), anemia (hemoglobin < 12g/dL), leukopenia (white-blood-cell count<4000/mm^3^), lymphopenia (lymphocyte count<1000/mm^3^) and neutropenia (neutrophil count<1500/mm^3^).

Paired MSG, serum and saliva samples from all participants were collected and stored according to the standard operations procedures of the HARMONICSS European-funded multi-centric protocol (H2020-SC1-2016; Grant Agreement No.: 731944). All available paired specimens collected during the past four years in the Department of Pathophysiology, School of Medicine, National and Kapodistrian University of Athens (NKUA), Greece were included in the study. Samples from all participants were collected after signed informed consent and the study was performed in the context of the HARMONICSS research protocol, which was approved by the Bioethics Committee of School of Medicine, NKUA, Greece on 20/07/2017.

### Evaluation of Serum and Saliva CXCL13 Levels

CXCL13 levels at serum and saliva samples were measured by a commercially available ELISA (sensitivity: 1 pg/ml; Abcam) according to manufacturer’s instructions.

### CXCL13 Expression and eGC Formation at the MSG Lesions

The expression of CXCL13 and the organization of MSG lymphoid infiltrates into eGCs were evaluated by a standard immunohistochemical technique (41) using antibodies against specific markers in serial sections. The presence of ectopic lymphoid structures in MSG lesions was evaluated by both hematoxylin and eosin staining and immunostaining in serial sections with antibodies recognizing specific markers of T, B and follicular dendritic cells, including CD3 (rabbit mAb, Cell-Marque, Rocklin, California, USA), CD20 [mouse monoclonal antibody (mAb) L26, Dako, Denmark)] and CD21 (rabbit mAb, EP3093, Abcam, Cambridge, UK), respectively, as well as other molecules that characterize eGCs, such as Bcl6 (mouse mAb PG-B6p, Dako) and AICDA (rabbit mAb ERP23436-45, Abcam). In pSS patients without available MSG biopsy specimen at the time of serum and saliva sampling, eGCs were recorded according to their presence in the diagnostic MSG biopsy. A monoclonal CXCL13 expression was detected by immunostaining with a rabbit monoclonal antibody (mAb) antibody (ERP23400-92, Abcam). Briefly, the immunohistochemical procedure was as follows: after deparaffinization, MSG sections (4μm) were blocked for endogenous peroxidase activity by a 20-min incubation in 0.5% H_2_O_2_ and antigens were retrieved by microwaving in Tris/EDTA solution, pH:9.0, for 15-min. To block non-specific antibody binding, slides were incubated in TBS buffer supplemented with 10% normal non-immune fetal bovine serum for 15-min, followed by overnight incubation at 4°C with primary antibodies and the application of the EnVision system (Dako) recognizing mouse and rabbit antibodies as second antibody and development system. Negative-controls used in each MSG tissue sample was staining with irrelevant isotype-matched antibodies and no addition of primary antibody, whereas staining of tonsil with all primary antibodies was routinely used as positive control in each experiment. CXCL13+ cells in MSG tissues were blindly counted field-by-field in each section (consisted of at least four MSG-lobules) by two independent observers (EKK, LC) and expressed as number of cells per mm^2^ of tissue.

### Statistical Analyses

Differences in CXCL13 serum or saliva levels among the various subgroups of pSS patients or pSS patients, sicca-controls, healthy individuals and non-SS NHL controls were analyzed by the non-parametric Kruskal-Wallis test and subsequent post-hoc Dunn’s multiple comparisons test to identify differences between specific pairs of groups. Significant differences in the CXCL13 serum or saliva levels between patients expressing or not various clinical, histological and serological markers were analyzed by the non-parametric Mann-Whitney test, and potential associations with continuous variables by Spearman’s rank correlation test. The over-time change of CXCL13 serum levels in sequential pre-lymphoma and lymphoma (on diagnosis) sera samples was analyzed by Wilcoxon’s matched pairs test. To evaluate disease features, including serum or saliva CXCL13 levels, associated with NHL development or high risk to develop NHL univariate analysis was performed. Categorical variables were compared by the Pearson chi-square or the Fisher exact test, when appropriate. To identify independent factors associated with NHL in SS, all variables associated with it with a p-value less than 0.1 in univariate analysis were further evaluated by multivariate binary logistic regression analysis with backward stepwise elimination. GraphPad Prism-5 (GraphPad Software, San Diego, CA, USA), Python 3.6 and SPSS-17 (Computing Resource Centre, Santa Monica, CA, USA) software were used. Statistical significance was defined as a p-value of less than 0.05 for all comparisons; p-values were 2-tailed. Only the statistically significant differences are reported.

## Results

### CXCL13 Expression in MSG Inflammatory Lesions Is Associated With Lesion Severity and CD21+-FDC Network

**Figure 1:**
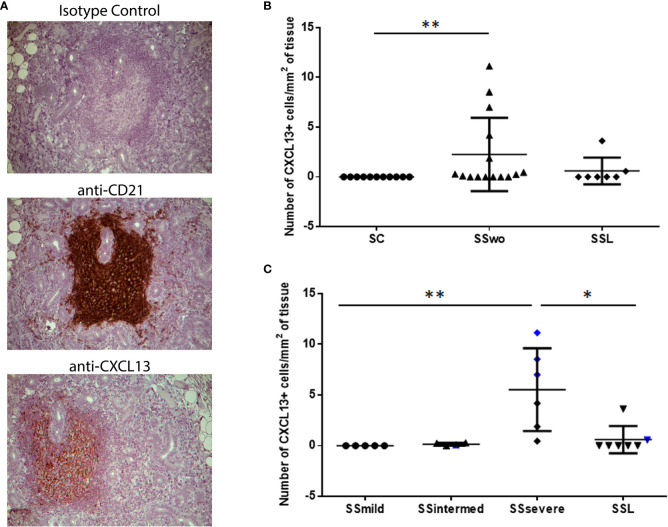
Levels of CXCL13 expression in the MSG tissues of pSS patients and sicca-complaining controls. **(A)** CXCL13+-cells are detected in areas of CD21+-FDC networks in the MSG tissues of pSS patients. Representative pictures of immunohistochemical staining with isotype antibody (negative control), anti-CD21 (CD21) and anti-CXCL13 (CXCL13) antibody in MSG sections from a pSS patient with severe infiltrates and germinal center formation are shown. Original magnification: x20. **(B)** Dot plot displaying the number of CXCL13+-cells per tissue area (mm^2^) in the MSG tissues of sicca-complaining controls (SC), pSS patients without evidence of NHL (SS) and pSS patients with NHL (SSL). **(C)** Dot plot displaying the number of CXCL13+-cells per tissue area (mm^2^) in the MSG tissues of the various subgroups of pSS patients without evidence of NHL, as classified according to lesion severity to those with mild (SSmild), intermediate (SSintermediate) and severe (SSsevere) infiltrates, as well as pSS patients with NHL (SSL). Counts in MSG tissues with ectopic germinal centers (eGCs) are designated by blue color. Comparisons in **(B, C)** were performed by the non-parametric Kruskal-Wallis test. P-values are designated by asterisks (*p < 0.05, **p < 0.01), whereas horizontal bars represent the mean value of the group. Only statistically significant associations are indicated.

Except one patient with intermediate infiltrates, who was at high risk for NHL development, CXCL13-positive cells were detected in areas of CD21+-FDCs networks within MSGs (**Figure 1A**). CXCL13-positive cells were observed within MSG inflammatory lesions in nine of fifteen pSS patients without evidence of NHL and in two of seven SSL patients who were immunohistochemically examined. CXCL13 staining was negative in all pSS patients with mild infiltrates (n=5) and all sicca-controls. The number of infiltrating CXCL13-positive cells per tissue area (mm^2^) was significantly different between pSS patients and sicca-controls (median, range: 0.25, 0.00-11.14 and 0.00, 0.00-0.00, respectively; p=0.0064). Among distinct pSS subgroups, the number of CXCL13-positive cells per tissue area was significantly higher in pSS patients with severe MSG infiltrates (median, range: 5.59, 0.46-11.14) compared to those with mild lesions (0.0, 0.0-0.0, p=0.0025) or SSL (0.00, 0.00-3.63, p=0.021) (**Figures 1B, C**), as well as in pSS patients at high risk to develop lymphoma compared to those at low risk (4.18, 0.28-8.54 and 0.00, 0.00-11.14, respectively; p=0.016).

### Serum, but Not Saliva, CXCL13 Levels Are Elevated in pSS Patients Compared to Controls and Associate With Histologic Features Indicative of Severe MSG Inflammatory Responses

**Figure 2:**
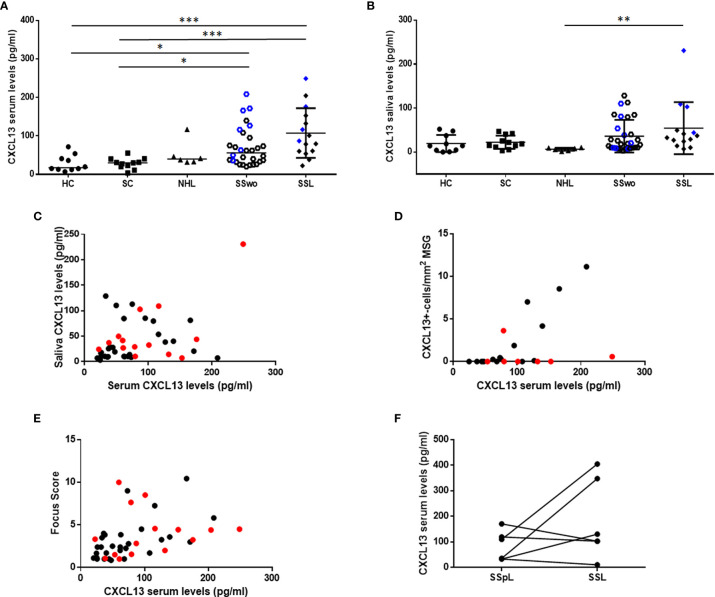
CXCL13 serum and saliva levels. **(A)** Dot plot displaying Kruskal-Wallis analysis of CXCL13 serum levels in healthy individuals (HC), sicca-complaining controls (SC), non-SS NHLs (NHL), pSS patients without evidence of NHL (SS) and pSS patients with NHL (SSL). **(B)** Dot plot representing Kruskal-Wallis analysis of CXCL13 saliva levels in healthy individuals (HC), sicca-complaining controls (SC), non-SS NHLs (NHL), pSS patients without evidence of NHL (SS) and pSS patients with NHL (SSL). P-values are designated by asterisks (*p < 0.05, **p < 0.01, ***p < 0.001), whereas horizontal bars represent the mean value of the group. Only statistically significant associations are indicated. CXCL13 serum and saliva levels from patients with ectopic germinal centers (eGCs) in the MSG infiltrates in panels **(A, B)** are highlighted by blue color. **(C–E)** Spearman’s rank correlation analysis of associations between: **(C)** serum and saliva CXCL13 levels (r=0.368, p=0.014), **(D)** serum CXCL13 levels and number of CXCL13+-cells per tissue area (mm^2^) in MSG tissues (r=0.534, p=0.011), **(E)** serum CXCL13 levels and biopsy focus score (r=0.644, p< 0.0001) in pSS patients. Red color designates samples obtained from pSS patients with NHLs. **(F)** Wilcoxon’s matched-pair analyses of CXCL13 levels in sequential serum samples from 6 pre-lymphoma pSS patients (SSpL) that transitioned to NHL (SSL) did not reveal any significant changes in CXCL13 expression levels before and on NHL diagnosis.

CXCL13 serum levels were significantly increased in pSS patients with or without NHL (median: 72.02 pg/ml and 87.00 pg/ml, respectively) compared to sicca-complaining controls (30.23 pg/ml; p=0.011 and p=0.0008 for pSS patients without or with NHL, respectively) and healthy individuals (17.56 pg/ml; p=0.012 and p=0.001, respectively) (**Figure 2A**). Although CXCL13 serum levels in patients with non-pSS associated NHLs (39.85 pg/ml) were lower than those with pSS patients (with or without NHL), they didn’t reach statistical significance. On the other hand, CXCL13 saliva levels were increased in SSL patients (34.92 pg/ml) compared to non-SS NHLs (6.92 pg/ml, p=0.0065), but were not significantly different from those in other study groups (18.57, 18.70 and 14.19 pg/ml in pSS, sicca-complaining controls and healthy individuals, respectively) (**Figure 2B**).

CXCL13 serum levels were positively associated with the saliva ones (r=0.368, p=0.014) (**Figure 2C**), and were correlated with the number of infiltrating CXCL13-positive cells per tissue area in the MSG lesions (r=0.534, p=0.011) (**Figure 2D**), a correlation that was further strengthened after excluding from the analysis pSS patients with NHL (r=0.797, p=0.0007). Again, CXCL13 serum, but not saliva, levels were found to correlate with MSG biopsy focus score (number of lymphocytic foci per 4 mm^2^ of tissue) (r=0.644, p< 0.0001) (**Figure 2E**). Serum CXCL13 levels were significantly higher in pSS patients with eGCs in autoimmune MSG lesions compared to those without (median 121.6 and 61.33 pg/ml, respectively, p=0.003). Among pSS patients with distinct MSG lesion severity, those with severe infiltrates were found to express significantly higher CXCL13 serum levels compared to those with mild lesions (105.7 pg/ml *vs* 34.63 pg/ml, respectively, p=0.012), whereas saliva levels did not differ significantly among the three pSS subgroups.

### CXCL13 Levels Correlate With Clinical and Laboratory Parameters Indicative of Adverse Outcome and/or NHL

**Figure 3:**
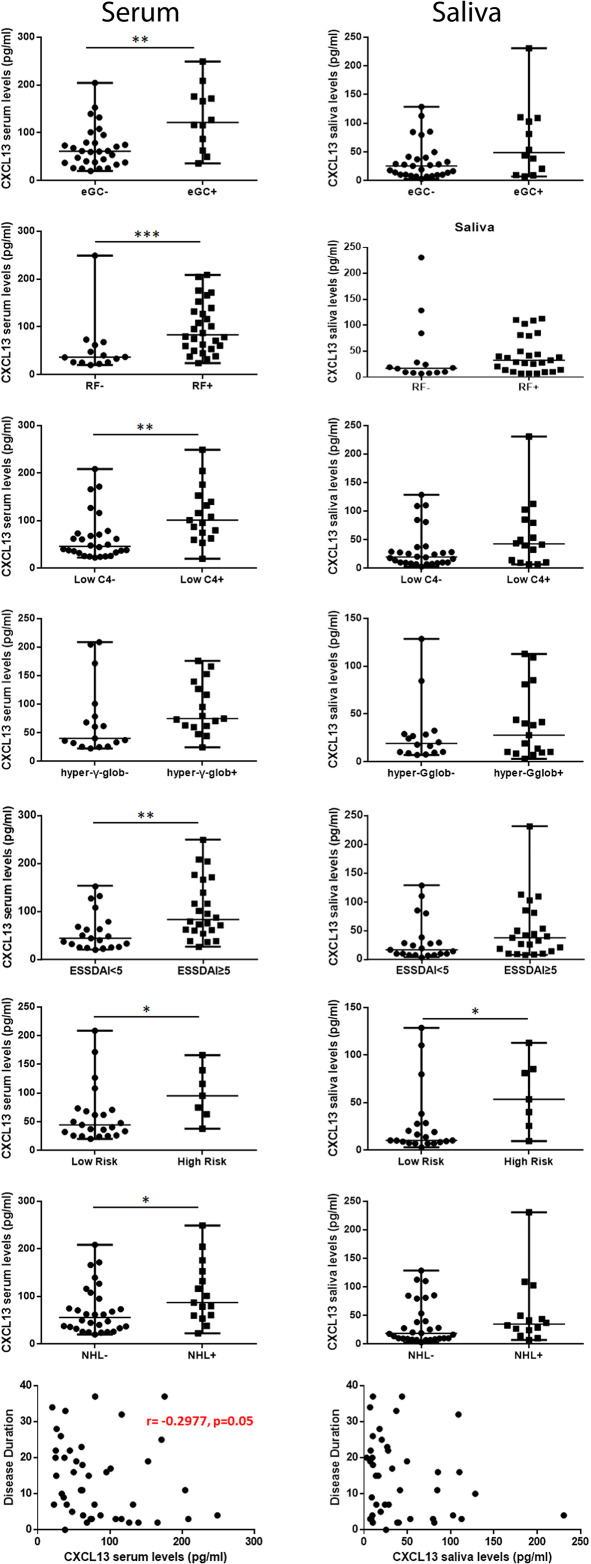
Association of CXCL13 serum and saliva levels with histologic, laboratory and clinical features. Mann-Whitney non-parametric analysis revealed that CXCL13 serum levels were significantly increased in pSS patients with ectopic germinal centers (eGCs) in the MSG infiltrates, presence of rheumatoid factor (RF), C4-hypocomplementemia (Low C4), hypergammaglobulinemia (hyper-γ-glob), high ESSDAI score (ESSDAI≥5), high risk to develop NHL (high risk) and NHL, whereas they were inversely correlated with disease duration. CXCL13 saliva levels were significantly increased in patients at high risk to develop NHL. P-values are designated by asterisks (*p < 0.05, **p < 0.01, ***p < 0.001), whereas horizontal bars represent the median value of the group.

The associations between CXCL13 serum and saliva levels and various histologic, clinical and laboratory parameters (summarized in **Table 2**) that have been associated with severe, systemic disease and/or NHL development have been examined. CXCL13 serum levels were significantly increased in pSS patients with rheumatoid factor (83.3 pg/ml *vs* 36.62 pg/ml in patients with rheumatoid factor *vs* those without, respectively, p=0.0009), hypocomplementemia (101.0 pg/ml *vs* 46.18 pg/ml, respectively, p=0.002), ESSDAI score≥5 (83.29 pg/ml *vs* 44.57 pg/ml, respectively, p=0.024) and NHL (87.00 pg/ml *vs* 55.87 pg/ml respectively, p=0.036), and marginally higher in pSS patients with hypergammaglobulinemia (75.0 pg/ml *vs* 40.26 pg/ml, respectively, p=0.073). Both CXCL13 serum and saliva levels were significantly increased in high risk pSS patients for NHL development compared to those in low risk (median serum concentration: 95.31 pg/ml *vs* 44.57 pg/ml, p: 0.025; median saliva concentration 53.56 pg/ml *vs* 10.31 pg/ml in patients at high and low risk, respectively, p= 0.019), whereas CXCL13 saliva levels were marginally higher in patients with high disease activity (37.23 pg/ml *vs* 16.55 pg/ml in patients with ESSDAI score≥5 *vs* those with ESSDAI<5, p=0.057). Lastly, CXCL13 serum levels were inversely correlated with disease duration (r=-0.2977, p=0.05) (**Figure 3**).

**Table 2:** Demographic, histologic, laboratory and clinical features of pSS patients that were evaluated for association with the serum, saliva or MSG levels of CXCL13 or NHL.

Disease features	Association with	
	CXCL13 levels	NHL
Age on sampling	X	X
Age on disease diagnosis	X	
Age on disease onset	X	
Disease duration	X	X
Focus score	X	X
Ectopic germinal centers	X	X
Salivary Gland enlargement	X	X
Rheumatoid factor	X	X
ANA autoantibodies	X	X
Ro/La autoantibodies	X	X
C3 hypocomplementenemia	X	X
C4 hypocomplementenemia	X	X
Cryoglobulinemia	X	X
Hyperglobulinemia	X	X
Leukopenia (at sampling)	X	X
Lymphopenia (at sampling)	X	X
Neutropenia (at sampling)	X	X
Monoclonal gamopathy	X	X
Anemia (at sampling)	X	X
Arthralgias/arthritis	X	X
Arthritis	X	X
Palpable purpura	X	X
Raynaud’s phenomenon	X	X
Vasculitis	X	X
Peripheral Neuropathy	X	X
Pulmonary involvement	X	X
Liver involvement	X	X
Kidney involvement	X	X
Splenomegaly	X	X
Lymphadenopathy	X	X
ESSDAI score	X	X
ESSDAI score ≥5	X	X
High-risk to develop NHL	X	
NHL	X	
Type of NHL (MALT)	X	
NHL location (*MSG vs other*)	X	

X mark is used to indicate the comparisons performed.

Since CXCL13 serum levels were found to correlate with various clinical parameters previously associated with the NHL prediction in pSS, we subsequently investigated whether CXCL13 levels in serum and/or saliva associate with NHL in pSS. Univariate analysis revealed that pSS-related NHL in our cohort significantly correlates with higher CXCL13 serum levels (p=0.036), age (p=0.007), disease duration (p=0.071), rheumatoid factor (p=0.04), hypocomplementemia (p=0.012), cryoglobulinemia (p=0.012), purpura (p=0.036), and high disease activity score (ESSDAI ≥5; p=0.052). Subsequently, the significance of these parameters (CXCL13 serum levels, age, disease duration, rheumatoid factor, hypocomplementemia, cryoglobulinemia, purpura, and high disease activity score) was further tested in multivariate analysis. Age (Beta Coefficient: 0.146, p=0.003) and hypocomplementemia (Beta Coefficient: -3.322, p=0.005) remained as independent parameters associated with NHL in multivariate analysis.

Finally, in an attempt to evaluate whether CXCL13 serum levels change upon transition to lymphoma, we estimated its levels in sequential sera of six pSS patients before and at NHL onset. Although the CXCL13 serum levels were not found to statistically change upon transition to lymphoma, we noticed a serial increase in half patients (**Figure 2F**).

## Discussion

Our findings confirm previously published data indicating that CXCL13 serum levels in pSS patients are elevated compared to sicca-complaining controls or healthy individuals and associate with the severity of MSGs infiltrates, their organization in eGCs and increased risk for NHL development (26, 27, 29). This is in agreement with the long-time known central role of CXCL13 in the recruitment of B cells in secondary lymphoid tissues, the trafficking of B and T follicular cells in GCs and their compartmentalization, and therefore, in B cell responses, antibody production, and lymphomagenesis (7, 9–11, 42, 43). Increased number of infiltrating CXCL13-positive cells was found in severe MSG lesions of pSS patients compared to SSL patients, as well as in high risk pSS patients for lymphoma development. Furthermore, despite the fact that the number of infiltrating CXCL13-positive cells correlate with respective serum levels, this correlation was further strengthened by the exclusion of SSL samples, whereas CXCL13 serum levels did not differ between pSS patients with or without NHL. These observations implicate CXCL13 in the progress of lymphomagenesis associated with the disease and most likely with the generation, survival, activation and/or expansion of autoreactive B cells within eGCs predisposing patients in NHL development (7, 10). In support of this, serum CXCL13 has been identified as a biomarker of GC activity and production of antibodies after vaccination (44, 45) and of systemic immune activation and disease activity in both infection and autoimmune diseases (14, 15, 18, 43, 46). In this context, the association of CXCL13 serum levels in pSS patients with clinical and laboratory markers of B cell activation, including rheumatoid factor, hypergammaglobulinemia, hypocomplementemia, and high disease activity was rather anticipated. Furthermore, elevated CXCL13 levels have been linked to prediction, presence, prognosis and/or therapeutic response of NHLs (47–51). Although our findings along with previous studies (27, 29), indicate that CXCL13 serum levels in pSS patients may associate with high risk to develop NHL, all studies failed to register CXCL13 serum levels as an independent lymphoma predictor. In the current study, we also evaluated CXCL13 serum levels in six patients with NHLs. Even though, CXCL13 serum levels in NHLs were lower than in SSLs, it did not reach statistical significance, due to the small sample size. Although these findings need to be confirmed in larger cohorts, CXCL13 serum levels before lymphoma onset did not change significantly upon transition to lymphoma, suggesting that this chemokine is upregulated before the clinical onset of NHL and therefore, it is implicated in earlier stages of lymphomagenesis.

An unanswered question is the origin of the elevated CXCL13 serum levels in pSS. FDCs and macrophages in liver, spleen and lymph nodes are considered as the major source of CXCL13 (9, 10). However, the correlation of CXCL13 serum levels with the number of CXCL13-positive cells within the MSG inflammatory lesions found in this study and with various histologic parameters, including the degree of MSG inflammation and the presence of eGCs, shown in this and previous studies (24, 26, 29), suggest that at least a part of the elevated CXCL13 serum levels in pSS patients arise from the affected salivary glands. This is in agreement with previous findings linking local and systemic autoimmune responses in pSS (33, 52, 53), as well as relevant findings in other autoimmune diseases suggesting that CXCL13 serum levels reflect the local inflammation in the affected organs (18, 54). On the other hand, we have previously observed that the number of infiltrating FDCs in MSG lesions of pSS patients decreases in more severe lesions (33). Though, there is no available data, since the secondary lymphoid organs have poorly studied in pSS, a possibility of migration of FDCs in regional or even distant lymph nodes, driving eventually the production of CXCL13, seems to be a reasonable explanation.

Intriguingly, CXCL13 saliva levels were not proved to be associated with disease characteristics although they were elevated in patients with eGCs in MSG autoimmune lesions and in high risk pSS patients for NHL development. This is in agreement with a previous study reporting that CXCL13 serum, but not saliva, levels may have a diagnostic utility in an Asian-Indian patient cohort (31). Although CXCL13 saliva levels correlate with serum levels, there is no association with the number of infiltrating CXCL13-positive cells or the extend of inflammatory lesions in MSGs of pSS patients. Thus, it paradoxically seems that CXCL13 saliva levels do not reflect its local production in MSG tissues, possibly due to rapid degradation by saliva proteases.

The major advantage of this study is the parallel evaluation of CXCL13 in paired serum, saliva and MSG tissues, allowing the cross-examination of associations with disease aspects. Although the size of study cohort is rather small, not permitting elaborated analyses, we found that CXCL13 serum, but not saliva, levels are associated with disease characteristics indicative of systemic active disease and lymphoma, supporting its role in disease pathogenesis. However, as previously reported (27, 29), we were unable to identify CXCL13 as an independent parameter associated with lymphoma development, a fact that hampers its use as a single molecular biomarker. On the other hand, CCLX13 might participate in combined scores of the activity of pSS, being an important laboratory element for the creation of clinically useful endpoints for the forthcoming therapeutic trials in pSS.

## Data Availability Statement

The raw data supporting the conclusions of this article will be made available by the authors, without undue reservation.

## Ethics Statement

The studies involving human participants were reviewed and approved by Bioethics Committee of School of Medicine, National and Kapodistrian University of Athens. The patients/participants provided their written informed consent to participate in this study.

## Author Contributions

EK designed and supervised the study, performed experiments, analyzed the data, and wrote the paper. LC performed experiments, selected and/or recruited the study participants, recorded and evaluated the clinical data, and participated in manuscript preparation. IS participated in the selection and/or recruitment of study participants, recorded and evaluated the clinical data. AG contributed in evaluation of clinical data, data analysis, and manuscript preparation, MV in study design and data analysis and AT in study design and supervision, as well as manuscript preparation. EK, AG, and LC had full access to all the data in the study and take responsibility for the integrity of the data and the accuracy of the data analysis. All authors contributed to the article and approved the submitted version.

## Funding

The research has been financed by grants of the Research Grant from the Greek Rheumatology Society and Professional Association of Rheumatologists and the European-funded multi-centric protocol “HARMONIzation and integrative analysis of regional, national and international Cohorts on primary Sjögren’s Syndrome (pSS) towards improved stratification, treatment and health policy making” (HARMONICSS; H2020-SC1-2016; Grant Agreement No.: 731944).

## Conflict of Interest

AT has received research grants from NOVARTIS, PFIZER, UCB, ABBVIE and GSK pharmaceutical companies, through the National and Kapodistrian University of Athens, outside the submitted work.

The remaining authors declare that the research was conducted in the absence of any commercial or financial relationships that could be construed as a potential conflict of interest.

## Publisher’s Note

All claims expressed in this article are solely those of the authors and do not necessarily represent those of their affiliated organizations, or those of the publisher, the editors and the reviewers. Any product that may be evaluated in this article, or claim that may be made by its manufacturer, is not guaranteed or endorsed by the publisher.

## Abbreviations

pSS, primary Sjögren’s syndrome; MSG, minor salivary glands; NHL, non-Hodgkin’s lymphoma; ESSDAI, EULAR SS disease activity index; SGE, salivary gland enlargement; eGC, ectopic germinal center, MALT, mucosa-associated lymphoid tissue; DLCBL, diffuse large B-cell lymphoma; CLL, chronic lymphocytic leukemia; FS, focus score.
